# Supplementary material for: The Adaptive Role of Carotenoids and Anthocyanins in Solanum lycopersicum Pigment Mutants under High Irradiance
Source: Cells. 2023 Nov 3;12(21):2569. doi: 10.3390/cells12212569 (PMC10650732; doi:10.3390/cells12212569)
Supplement: Supplementary file 1 [file cells-12-02569-s001.zip › cells-2658838-supplementary.pdf]

Table S1. Primers for genes.

№	Gene Bank ID	Gene Description	Gene	Primer 5′-3′	
				forward	reverse
1	NM_001247346.1	Damage DNA binding protein 1	*DDB1*	ACTACGTGGTTACGGCTCAC	TGGCACATCTAACATAGGCTGTAA
2	NM_001374394.1	Anthocyanidin synthase	*ANS*	TCTCAATTCCCACCTCGCAC	ACTTTGCGCTCAGCAAGAAC
3	NM_001247883.2	Phytoene synthase	*PSY*	CTCCATCTGGAGAACGGACG	CAACAAGCCCAAATTCCCCG
4	NM_001247104.2	Chalcone synthase	*CHS*	CCGTGAACCCAGTGAATCTC	CTCACGTAGGTGTCCGTCAAT
5	NM_001247891.2	Transcription factor elongated hypocotyl 5	*HY5*	AAGCTCAACCATCAGCTGGG	CCTTCACCCTTGCTTCCAGA
6	NM_001308008.1	Transcription factor phytochrome interacting factor 4	*PIF4*	GAGTTGTTGTGGCGAAACGG	TAGCCACGCGACAGTTTCAT
7	NM_001247118.2	E3 ubiquitin-protein ligase	*COP1*	TGAACCTGCAGATGCACACT	AATCAACACTCCAAGCCCGT
8	XM_004249510.4	Phenylalanine ammonia-lyase 1	*PAL1*	TGCAGCTCCAACTACCCTTT	ATTCGTCCTCGAAAGCTCCA
9	XM_004242121.4	Flavonol synthase	*FLS*	CGACAGAAGCTGGGATTTCCT	GGATCCATTCGCCGTCCTTT
10	NM_001247219.2	De-etiolated1	*DET1*	AAGCTGGCAGCACAGATGG	CTGATGCCTGCAGAAACAAGG
11	A0A3Q7F8W6	Tubulin alpha chain	*TUB1*	ACAACTTTGCCCGTGGACAT	TGCTCAAGAAGGGAGTGGGT
